# Evaluating the empirical evidence for the two-stage-model of infant imitation. A commentary on Paulus, Hunnius, Vissers, and Bekkering (2011)

**DOI:** 10.3389/fpsyg.2012.00512

**Published:** 2012-11-21

**Authors:** David Buttelmann, Norbert Zmyj

**Affiliations:** ^1^Research Group “Kleinkindforschung in Thueringen,” University of ErfurtErfurt, Germany; ^2^Ruhr-Universität BochumBochum, Germany

**A commentary on**

**Bridging the gap between the other and me: the functional role of motor resonance and action effects in infants' imitation**

by Paulus, M., Hunnius, S., Vissers, M., and Bekkering, H. (2011). Dev. Sci. 14, 901–910.

It has been proposed that infants selectively imitate based on a rational evaluation of the observed action (“rational-imiation-account,” Gergely et al., 2002). This view has been rejected by Paulus et al. (2011a) proposed a “two-stage model of infants” ability to imitate observed action-effect contingencies'. They claimed that infant imitation depends on (1) similarity between the infants' and the model's body posture; and (2) the presence of action effects (“two-stage-model”). However, there are alternative explanations for Paulus et al.'s (2011a) results that limit significant developmental conclusions regarding infant imitation.

In the task introduced by Meltzoff (1988), infants were presented with a model who illuminated a lamp by an unusual means, i.e., her head (in the following referred to as “head-touch”), instead of a usual means, i.e., her hands (*hands-free* condition, Gergely et al., 2002). According to the rational-imitation-account, infants consider others to always use the most efficient means to achieve a goal. Since the model used her head although her hands were free, infants—whose hands were never constrained—imitated the head-touch to explore the benefit of this action. In contrast, they imitated the head-touch less often when the model had to use her head because her hands were occupied holding a blanket (*hands-occupied* condition, Gergely et al., 2002). In this condition, infants regarded the head-touch as the most effective means given the model's constraints and, since infants' constraints differed, not worth copying (Gergely and Csibra, 2003, p. 291).

Paulus and colleagues (2011a) reported four new variations on this task.

In the *button* condition—adapted from a recent study (Paulus et al., 2011b)—the blanket was held around the model's shoulders by a large red button instead of her hands. Thus, the model's hands were free but hidden underneath the blanket. Infants imitated the head-touch in this condition less often than in the hands-free condition. The authors concluded that infants' tendency to imitate depended on the degree of resonance between the observed action and the infants' motor repertoire. This motor resonance is stronger when the model's action is in the infants' motor repertoire. Since infants cannot perform the head-touch without putting their hands next to the lamp, there is not enough resonance with the model's action when her hands are crossed in front of her upper body (Paulus et al., 2011b, p. 1049). However, the manipulation in the button condition is problematic for two reasons. First, although the model highlighted that she could use her hands before demonstrating the head-touch (e.g., by re-arranging her chair), it is unclear whether infants remembered these demonstrations when the model performed the subsequent head-touch. Even if they did, it is possible they conceived the two actions as independent intentional events (Baldwin et al., 2001). Relatedly, the model's hands were not visible while she performed the head-touch. Thus, infants might have seen her hands as being occupied. Since her hands were hidden underneath the blanket, this demonstration resembled the demonstration in Gergely et al.'s (2002) hands-occupied condition in which infants rarely imitated the head-touch. Second, it is unclear whether infants understand the function of buttons. Although they have probably seen buttons on jackets, infants do not necessarily understand their function, especially in the context of a blanket being wrapped around one's upper body.

In two of the new conditions, the *head-only* and the *head-effect* conditions, the blanket was held by a salient button as in the button condition when the model performed the head-touch. In the head-only condition, the model demonstrated that the lamp did not light up when she initially used her hand, but that it did when she subsequently used her head. The head-effect condition was identical, except that no hand action was demonstrated. As in the button condition, significantly fewer infants copied the head-touch in these conditions compared to the hands-free condition. The authors took this as evidence for the two-stage-model: if the model's action does not lead to motor resonance then the tendency to imitate is reduced. This mechanism works even if logical evaluation suggests the contrary (e.g., head-only: “the lamp can only be illuminated using the head—not the hand”). However, the same aforementioned problems apply here: we do not know whether infants (1) perceive the hands as being free; and (2) understand the function of buttons. Noteworthy, the lamp was switched off when it was handed to infants during the response phase in these two conditions. Infants might have perceived the apparatus as broken when they first tested it with their hands and, therefore, did not imitate the head-touch. Additionally, the rational-imitation-account makes no clear predictions for the head-only condition. The model's constraint (i.e., failing to illuminate the lamp with the hand) is identical to the infants' constraint. This differs from the original conditions in which either both infants and model were unconstrained or the model was constrained but infants were free.

In the *no-effect* condition, the procedure was identical to the hands-free condition except that the switched-off lamp did not light up when the model performed the head-touch. The two-stage-model predicts that infants do not imitate the head-touch since “a modeled action that does not lead to a salient effect would be unlikely to be imitated” (Paulus et al., 2011a, p. 906). In contrast, Paulus et al. claimed that the rational-imitation-account predicts that infants imitate the head-touch because the action, the constraints, and the goal were comparable to the hands-free condition. In fact, fewer infants imitated the head-touch in the no-effect condition than in the hands-free condition, which was interpreted as evidence for the two-stage-model. However, it is questionable whether the goal (in the sense of “external result,” Gergely and Csibra, 2003) is identical in the hands-free and the no-effect condition. Whereas the goal in the hands-free condition is the illuminated lamp, the goal in the no-effect condition is making contact with the lamp. Since these goals differ, it is debatable whether the rational-imitation-account would predict the same imitative behaviour in both conditions.

Taken together, a closer look at Paulus et al.'s (2011a) study revealed that alternative explanations that are in line with the rational-imitation-account hold for infants' performance in the new conditions. Additionally, we showed that the predictions for these new conditions are not as unambiguous as proposed by the authors. We acknowledge, however, that Paulus et al. (2011a) earned the merit of highlighting potential confounding variables in imitation tasks (e.g., the model's body posture). Further research on infants' selective imitation should conform with the idea of carefully considering alternative explanations of the experimental manipulations.
